# Rapid expansion and extinction of antibiotic resistance mutations during treatment of acute bacterial respiratory infections

**DOI:** 10.1038/s41467-022-28188-w

**Published:** 2022-03-09

**Authors:** Hattie Chung, Christina Merakou, Matthew M. Schaefers, Kelly B. Flett, Sarah Martini, Roger Lu, Jennifer A. Blumenthal, Shanice S. Webster, Ashley R. Cross, Roy Al Ahmar, Erin Halpin, Michelle Anderson, Nicholas S. Moore, Eric C. Snesrud, Hongwei D. Yu, Joanna B. Goldberg, George A. O’Toole, Patrick McGann, Jason A. Stam, Mary Hinkle, Alexander J. McAdam, Roy Kishony, Gregory P. Priebe

**Affiliations:** 1 Department of Systems Biology, Harvard Medical School, Boston, MA USA; 2 Department of Anesthesiology, Critical Care and Pain Medicine, Boston Children’s Hospital, Boston, MA USA; 3 Department of Anaesthesia, Harvard Medical School, Boston, MA USA; 4 Department of Microbiology and Immunology, Geisel School of Medicine at Dartmouth, Hanover, NH USA; 5 Division of Pulmonary, Allergy & Immunology, Cystic Fibrosis and Sleep, Emory Children’s Center for Cystic Fibrosis and Airway Disease Research, Department of Pediatrics, Emory University School of Medicine, Atlanta, GA USA; 6 Department of Biomedical Science, Joan C. Edwards School of Medicine at Marshall University, Huntington, WV USA; 7 Harvard Medical School, Boston, MA USA; 8 Walter Reed Army Institute of Research, Silver Spring, MD USA; 9 Department of Pediatrics, Joan C. Edwards School of Medicine at Marshall University, Huntington, WV USA; 10 Department of Laboratory Medicine, Boston Children’s Hospital, Boston, MA USA; 11 Faculty of Biology and Computer Science, Technion—Israel Institute of Technology, Haifa, Israel; 12 Present Address: The Broad Institute of MIT and Harvard, Cambridge, MA USA; 13 Present Address: Novant Health Eastover Pediatrics, Charlotte, NC USA; 14 Present Address: Winchester Hospital, Winchester, MA USA; 15 Present Address: Becton Dickinson, Franklin Lakes, NJ USA

**Keywords:** Antibiotics, Molecular evolution, Infectious-disease diagnostics

## Abstract

Acute bacterial infections are often treated empirically, with the choice of antibiotic therapy updated during treatment. The effects of such rapid antibiotic switching on the evolution of antibiotic resistance in individual patients are poorly understood. Here we find that low-frequency antibiotic resistance mutations emerge, contract, and even go to extinction within days of changes in therapy. We analyzed *Pseudomonas aeruginosa* populations in sputum samples collected serially from 7 mechanically ventilated patients at the onset of respiratory infection. Combining short- and long-read sequencing and resistance phenotyping of 420 isolates revealed that while new infections are near-clonal, reflecting a recent colonization bottleneck, resistance mutations could emerge at low frequencies within days of therapy. We then measured the in vivo frequencies of select resistance mutations in intact sputum samples with resistance-targeted deep amplicon sequencing (RETRA-Seq), which revealed that rare resistance mutations not detected by clinically used culture-based methods can increase by nearly 40-fold over 5–12 days in response to antibiotic changes. Conversely, mutations conferring resistance to antibiotics not administered diminish and even go to extinction. Our results underscore how therapy choice shapes the dynamics of low-frequency resistance mutations at short time scales, and the findings provide a possibility for driving resistance mutations to extinction during early stages of infection by designing patient-specific antibiotic cycling strategies informed by deep genomic surveillance.

## Abstract (web summary)

It remains unclear how rapid antibiotic switching affects the evolution of antibiotic resistance in individual patients. Here, Chung et al. combine short- and long-read sequencing and resistance phenotyping of 420 serial isolates of *Pseudomonas aeruginosa* collected from the onset of respiratory infection, and show that rare resistance mutations can increase by nearly 40-fold over 5–12 days in response to antibiotic changes, while mutations conferring resistance to antibiotics not administered diminish and even go to extinction.

## Introduction

Antibiotic treatment selects for resistance mutations, posing a major threat to effective treatment of bacterial infections^1^. The selection of resistance mutations during chronic infections as a result of antibiotic treatment over months to years is well-known^2–9^. However, it is not well-understood how short-term changes in antibiotic therapy affect the dynamics of resistance mutations in acute infections, especially in a newly colonizing infection that is thought to start from a clonal population^10^.

Emerging resistance is of particular concern in the treatment of acute respiratory tract infections that are common in intensive care units (ICUs) worldwide, particularly in mechanically ventilated patients who are at high risk for ventilator-associated pneumonia (VAP), septic shock, and infection-associated mortality^11–15^. VAP and other lower respiratory tract infections are of major concern in the SARS-CoV-2 pandemic given the large number of hospitalized COVID-19 patients requiring ventilation^16–19^. *Pseudomonas aeruginosa* is one of the most common bacterial pathogens causing respiratory infections in ventilated patients, and is associated with increased mortality and low treatment efficacy due to the high rates of antibiotic resistance that can occur within days of antibiotic treatment^10,20–26^.

Shallow profiling of pathogen populations using cultured isolates have shown that the frequencies of antibiotic resistance mutations can fluctuate over days to weeks during infection^10,27^, but whether these changes reflect drift, sampling bias, or treatment-induced selection at short timescales is unknown. Current clinical methods for detecting resistance variants are largely culture-based, where isolates with visually distinct morphology (by size, shape, and color) are selected for resistance phenotyping. However, these methods are susceptible to bias from culture-based growth and are limited in their sampling resolution, especially for detecting low-frequency mutations. While molecular surveillance methods such as rapid PCR tests and real-time genome sequencing can identify the presence of known resistance genes^28–31^, e.g., efflux pumps, to identify resistant strains, they are not suitable for monitoring within-population pathogen diversity. Furthermore, it is not well-understood whether resistance mutations can contract and be reversed during the course of treatment in acute infections. A molecular, culture-free diagnostic could determine the role of low-frequency resistance variants at short time scales, and possibly inform which antibiotics should be avoided.

Here we combine whole-genome sequencing with resistance-targeted deep amplicon sequencing (RETRA-Seq) to show that resistance mutations, either pre-existing or de novo, expand and contract rapidly within days of changes in therapy. By conducting a deep sampling study of *P. aeruginosa* populations, and using long-read sequencing to construct patient-specific reference genomes in order to maximize the detection of within-population mutations, we construct a high-resolution view of pathogen evolution during acute respiratory infection. We then relate how changes in empirically administered antibiotics impact resistance in individual patients, and discover that resistance mutation frequencies change within days, depending on the duration and type of antibiotic therapy.

## Results

### Prospective study of *P. aeruginosa* populations during acute respiratory infections

**Fig. 1:**
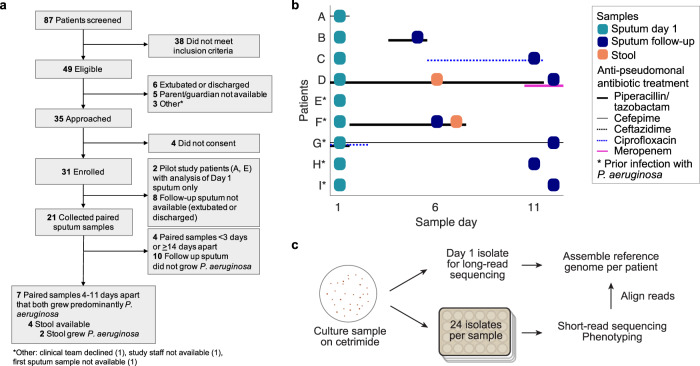
Prospective study of *P. aeruginosa* populations from mechanically ventilated patients during acute respiratory tract infection. **a** Prospective study design describing the enrollment strategy of mechanically ventilated patients in the ICU. Of 87 patients screened, 49 eligible patients were identified, from which 31 consented to enrollment. We focused on 2 pilot patients sampled at only day 1, and 7 patients sampled serially across 4–11 days that exhibited predominant *P. aeruginosa* growth in both samples. **b** Sampling sputum and stool across patients (*y*-axis) over time (*x*-axis) from the onset of symptoms. Day 1 sputum sample (teal box) were collected in all patients, and a follow-up sputum (dark blue box) were collected in 7 patients between day 5 and day 12, i.e. 4–11 days after day 1. Stool samples (brown box) with confirmed *P. aeruginosa* growth were collected in 2 patients. Asterisk: patients with prior *P. aeruginosa* infection. Treatment with anti-pseudomonal antibiotics are indicated by horizontal lines: piperacillin/tazobactam (weighted black), cefepime (thin black), ceftazidime (dotted black), ciprofloxacin (dotted blue), meropenem (weighted pink). **c** Samples (sputum or stool) were cultured on cetrimide agar in serial dilutions. A single isolate from day 1 sputum of each patient was used to construct a patient-specific reference genome using long-read sequencing. From each sample, 24 isolates were randomly selected for short-read whole-genome sequencing and phenotyping.

We conducted a prospective study of mechanically ventilated patients with clinical evidence of acute respiratory tract infection in the pediatric or cardiac intensive care unit at Boston Children’s Hospital. Eighty-seven patients were screened to identify 49 patients that met the inclusion criteria, of which 31 patients consented to enrollment (Fig. 1a; Methods). Endotracheal or tracheal aspirates (referred here throughout as sputum samples) were collected at the onset of symptoms (‘sputum day 1’), with serial samples (‘sputum follow-up’) collected when possible. We first conducted a small pilot study to assess the genomic diversity of *P. aeruginosa* in two patients, patients A and E*, who were sampled only at day 1. After confirming population growth and detectable diversity, we focused on studying short-term infection dynamics in 7 patients whose serial samples were collected 4–11 days after day 1 and exhibited *P. aeruginosa* growth at both time points as the predominant pathogen (Fig. 1b; Supplementary Fig. 1; Methods; Supplementary Table 1). In addition, as GI tract carriage is thought to be a source of intrapatient infection^32^, stool was also collected if available, of which only 2 of 4 available samples exhibited *P. aeruginosa* growth. Among the 9 patients (7 serially sampled and 2 pilot study patients), 4 had no history of *P. aeruginosa* infection (patients A-D) while 5 had a documented history of prior *P. aeruginosa* infection (denoted by an asterisk, patients E*-I*). In total, we collected 18 sputum and 2 stool samples across 9 patients from the onset of infection.

### Maximizing the detection of genomic diversity by constructing patient-specific reference genomes

To capture the full extent of genomic diversity in pathogens, we used both long-read and short-read sequencing to characterize the *P. aeruginosa* populations in each patient. *P. aeruginosa* has a flexible pangenome with variations in gene content across strains by up to 50%^33^. A poor choice in the reference genome would impact the alignment rate of short reads and therefore, the fraction of usable reads for identifying within-patient polymorphisms. We thus assembled a complete patient-specific reference genome using long-read sequencing of a single *P. aeruginosa* colony per patient^10,34,35^ (Fig. 1c), which supported that each patient was infected with a unique strain based on gene content (Supplementary Fig. 2a; Methods). To capture within-patient diversity, we collected 24 additional cultured isolates from each sputum or stool (*n* = 420 total), sequenced their whole genomes with short reads, and aligned these reads to the patient-specific reference genomes (average alignment rate > 99%, Supplementary Fig. 2b) in order to identify within-patient single nucleotide polymorphisms (SNPs) and short insertions and deletions (indels) (Supplementary Fig. 2c; Supplementary Data 1). We then used the within-patient variants (SNPs and indels) to construct patient-specific phylogenies of *P. aeruginosa* populations and to infer the most recent common ancestor (MRCA) in each patient (Methods).

### New infections start with clonal founders

**Fig. 2:**
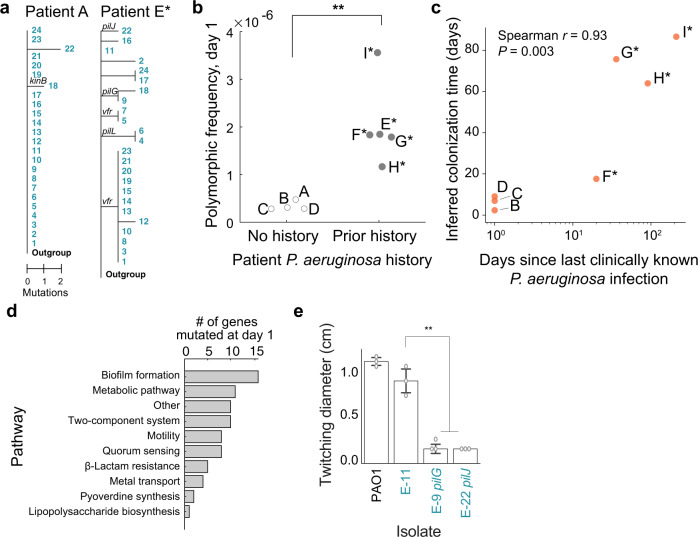
Patient infection history impacts genomic diversity of *P. aeruginosa* at the onset of infection. **a** Phylogenetic trees of *P. aeruginosa* populations in pilot patients A and E* rooted with an Outgroup (Methods). Numbers (rows) correspond to tree leaves (teal) representing an isolate. Scale: mutational events (single nucleotide polymorphisms (SNPs) and indels) from the most recent common ancestor (MRCA) in each patient. Select branches are labeled with mutated genes. **b** Comparing the initial pathogen diversity of patients (dots) based on prior history of *P. aeruginosa* infection. Frequency of polymorphic loci (*y*-axis; calculated as number of unique SNP or indel positions divided by genome size) in patients with no prior *P. aeruginosa* history vs. in patients with clinically documented infection history (*x*-axis). Significance: *P* = 0.007, two-sided *t*-test. **c** Relation between the estimated colonization time of the pathogen in each patient (days, *y*-axis; Methods) and time to the last clinically documented infection (days, *x*-axis). Spearman correlation (two-sided), *r* = 0.93, *P* = 0.003. **d** Pathways (*y*-axis) found in mutations of coding regions at day 1 (*x*-axis) across all patients. **e** Altered twitching phenotype in isolates with single point mutations in genes of the *pil* locus. Isolates (*x*-axis) assayed for twitching diameter (cm, *y*-axis; Methods), from left to right: PAO1 reference strain, E-11 isogenic control, E-9 singleton *pilG* mutant, E-22 *pilJ* singleton mutant. Each assay was conducted across 3 technical replicates (dots), representative of 3 biologically independent replicates. Bars show median; error bars, standard error (s.e.). Significance: Tukey’s multiple comparisons test (E-11 vs. E-9, *P* = 0.001; E-11 vs. E-22, *P* = 0.001; adjusted *P-*values).

The diversity of pathogens at the onset of infection depended on the infection history of patients. In the case of a presumed new infection in patient A, the population was nearly clonal, which was consistent with a recent colonization by a single founder^36–38^ (Fig. 2a, left). In contrast, the day 1 population was polymorphic in patient E*, who had a documented history of *P. aeruginosa* infection (Fig. 2a, right). Testing whether initial pathogen diversity—defined as the frequency of polymorphisms at day 1—differed by infection history across patients indeed revealed that patients with prior infection had higher initial diversity (Fig. 2b; *P* = 0.007, two-sided *t*-test). Suspecting that populations from prior infections were maintained in these patients, we compared the inferred colonization time of each patient based on genomic data (Methods) to the time since the last clinically documented *P. aeruginosa* infection, which were significantly associated (Fig. 2b; Spearman *r* = 0.93, *P* = 0.003), providing evidence that pathogen reservoirs were maintained between symptomatic episodes that resembles a chronic infection^6,7^. Consistently, mutations at day 1 were found in genes and pathways important for pathogen colonization^6^, such as biofilm formation (Fig. 2d; Supplementary Data 2) and impairment in motility (Fig. 2e, *P* < 0.0001). Altogether, these results show that new infections are colonized by a single clonal founder, and that once colonized, pathogen reservoirs can be maintained in patients between symptomatic episodes that resembles subchronic infection.

### Mutations that alter clinical phenotypes are accrued over days

**Fig. 3:**
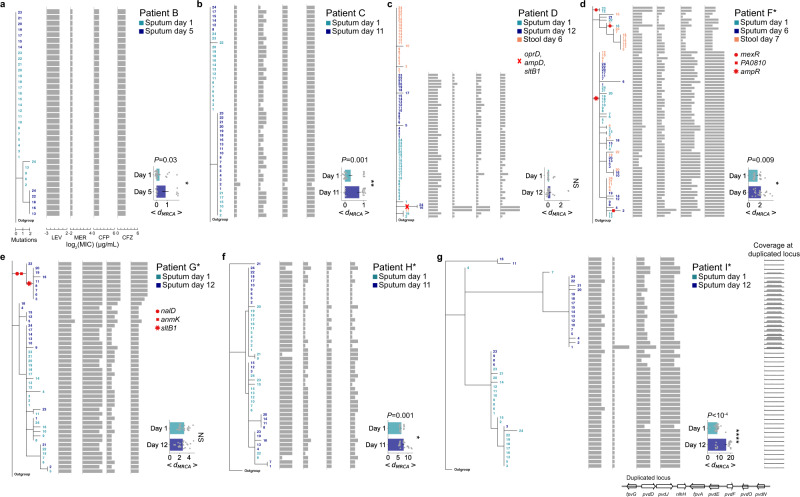
Phylogenetic analyses of *P. aeruginosa* populations within each patient and their corresponding antibiotic resistance profiles. **a–g** Left: Phylogenetic trees of *P. aeruginosa* populations in serially sampled patients: patient B (**a**), patient C (**b**), patient D (**c**), patient F* (**d**), patient G* (**e**), patient H* (**f**), patient I* (**g**). Numbers (rows) correspond to tree leaves that represent an isolate (day 1 sputum in teal, follow-up sputum in dark blue, stool in brown). Scale: mutational events (single nucleotide polymorphisms (SNPs) and indels) from the most recent common ancestor (MRCA) in each patient. A subset of branches associated with antibiotic resistance are marked with red symbols. Middle: Antibiotic resistance profiles (horizontal gray bars) in units of minimum inhibitory concentration (*log*_2_(MIC); µg/mL) of individual isolates (rows) aligned to the isolate’s position on the tree, shown for levofloxacin (LEV), meropenem (MER), cefepime (CFP), and ceftazidime (CFZ). Right: distance to the MRCA (<*d*_*MRCA*_>, *x*-axis) of isolates (gray dots) at each time point (*y*-axis, days of infection). Mean (horizontal bars) and standard error (error bars) calculated over *n* = 24 biologically independent isolates per sputum or stool sample (exception: *n* = 12 isolates in Day 5 sputum of patient B (**a**)). Significance, one-tailed permutation test: *P* = 0.03 (**a**), *P* = 0.001 (**b**), *P* = 0.009 (**d**), *P* = 0.001 (**f**), *P* < 10^−4^ (**g**). NS not significant. **g** Far right, bottom: schematic showing the relative copy number (*y*-axis) of a ~34 kb duplicated chromosomal region (*x*-axis) that encodes, among others, genes of the pyoverdine pathway (bottom).

Pathogen populations diversified within all patients by the emergence of single point mutations. In most patients, mutations accumulated significantly over days as quantified by the increase in distance to the most recent common ancestor (*d*_*MRCA*_; Fig. 3, horizontal bar plots insets, permutation test of <*d*_*MRCA*_>; Methods), although the frequencies of individual mutations could both increase or decrease (Supplementary Fig. 3a). Notably, stool and sputum populations within each patient, where observed, were indistinguishable (Fig. 3c, d), indicating either gut carriage as the source of respiratory colonization^10,32^, or more simply, that stool samples reflect the passage of ingested sputum through the gastrointestinal tract.

To assess whether mutations could reflect diversifying selection, we characterized clinically relevant phenotypes of a subset of non-synonymous mutations that increased in frequency (Supplementary Fig. 3a) or those that occurred recurrently in genes, which we interpret as under selection^2,3,5,8^ (Supplementary Fig. 3b). To estimate a genotype-phenotype relation, we focused on singleton mutations and compared isolates with and without only that mutation that were otherwise genetically identical, i.e., comparing mutants to estimated isogenic controls (based on >99% alignment rate of isolate genomes, Supplementary Fig. 2b). Point mutations impacted a wide range of clinically important phenotypes, including those in *wbpL* and *wzy* that altered lipopolysaccharide (LPS) and O-antigen presentation thereby affecting sensitivity to human serum (Supplementary Fig. 3c–f; Methods), and those in biofilm-related genes encoding BifA and KinB that impacted swarming, biofilm formation, and alginate production (Supplementary Fig. 3g–k). Altogether, our findings show that the evolution of *P. aeruginosa* over days leads to the diversification of clinically important phenotypes.

### Measuring the in vivo frequencies of resistance mutations

We identified mutations associated with resistance by mapping the antibiotic resistance profiles of isolates to their genomes (Fig. 3, red symbols; Supplementary Fig. 4). The frequencies of these mutations changed across days, based on cultured isolates, with some resistant mutants observed only in later time points. For example, *nalD* (a repressor of MexAB-OprM^39^), *anmK* (involved in peptidoglycan recycling^40^), and *sltB1* (a lytic transglycosylase^41^) were found in a sublineage of patient G* that appeared to emerge after day 1 (Fig. 3e). Another set of linked mutations—*oprD*, *ampD*, and *sltB1* in patient D—conferring resistance to meropenem and ceftazidime (Fig. 3c) were found at low frequencies in only the second time point.

**Fig. 4:**
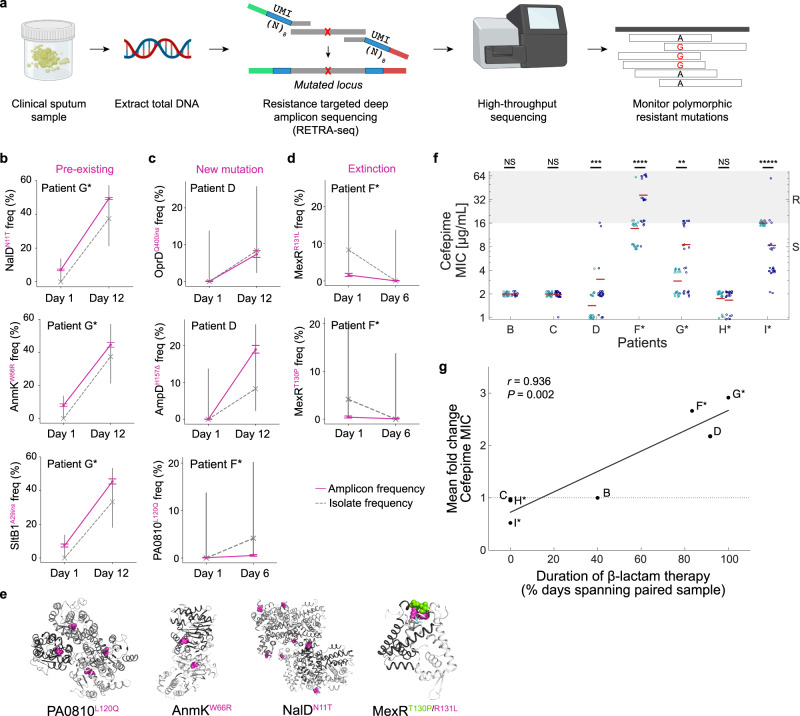
Treatment-associated dynamics of low-frequency resistance mutations. **a** Workflow of resistance-targeted deep amplicon sequencing (RETRA-Seq) as a diagnostic for monitoring resistance mutation frequencies. Total DNA is extracted from clinical sputum and prepared as sequencing libraries via PCR using primers that contain sequencing adapters (green, red) and unique molecular identifiers (UMIs; blue) composed of 8 degenerate nucleotides (N). Amplicon libraries are sequenced on a next-generation sequencing platform and aligned to a reference genome to determine polymorphic frequencies. Images created with BioRender.com. **b**–**d** Mutation frequencies (*y*-axis) across time (*x*-axis) of select resistance loci within each patient, measured by RETRA-Seq (solid pink) and by the fraction of culture-based isolates (dashed gray). Axis labels (*y*-axis) indicate the gene name and the mutation type (pink superscript): non-synonymous substitution, insertion (*ins*), or deletion (Δ). Error bars: Wilson Score interval of UMI counts (amplicon sequencing) or discrete counts (isolate sampling; Methods). Three types of changes in resistance frequencies: expansion of pre-existing mutations that were undetected by culture-based assay (**b**), expansion of presumed de novo mutations (**c**), and extinction of mutations (**d**). **e** Select non-synonymous mapped on protein structures of homologs of PA0810 (Protein Data Bank ID: 3UMC), AnmK (3QBW), NalD (5DAJ), and MexR (3ECH). Shades of gray indicate distinct monomers and pink/green spheres indicate mutated residues. **f** Distribution of cefepime MIC (µg/mL; *y*-axis) in individual isolates (dots) from day 1 (teal) and follow-up (dark blue) sputum samples, with mean value (horizontal read line). Ranges of resistance/intermediate susceptibility (R; gray) and sensitive (S; white) shown on the right and by background color, according to breakpoints defined by the Clinical Laboratory Standards Institute (CLSI). Significance (two-sided Mann–Whitney test): *P* = 7.5 × 10^−4^ (patient D), *P* = 2.2 × 10^−5^ (patient F), *P* = 0.004 (patient G), *P* < 10^−5^ (patient I). NS – not significant. **g** Relationship between cefepime resistance and clinical history of patient therapy. Fold change in mean cefepime MIC (*y*-axis) vs. the duration of β-lactam antibiotics administered to each patient during the study period (*x*-axis), shown for serially sampled patients (dots). Pearson’s correlation (two-sided), *r* = 0.936, *P* = 0.002.

To accurately capture the dynamics of resistance mutations in patients, without culture-based growth bias, we designed a scheme to measure the mutation frequencies directly from intact sputum samples by developing “resistance-targeted deep amplicon sequencing” (RETRA-Seq), in which we amplify the mutated loci from total DNA extracted from sputum for deep amplicon sequencing (Fig. 4a). In order to control for amplification bias and reliably measure the number of unique genomes across thousands of single cells that correspond to each allele, we incorporate unique molecular identifiers (UMIs) in the primers, and sequence at a saturating depth such that allele frequencies are resolved to the sequencing error rate (Supplementary Fig. 5; Supplementary Data 3; Methods).

RETRA-Seq of select resistance mutations (Fig. 3c-e, red symbols on branches of trees) revealed three types of in vivo mutation dynamics: (i) ‘pre-existing’ mutations that expanded from low frequencies at day 1 that were initially undetected by culture-based assay, (ii) presumed ‘de novo’ mutations within sequencing error, and (iii) mutations that went to ‘extinction’ (Fig. 4b–d). Some of these mutations impacted key residues at the interface of multimers, suggesting a loss-of-function (Fig. 4e). The observed magnitudes of expansions that occurred in vivo were striking: for instance, pre-existing mutations in *nalD, anmK*, and *sltB1* started at 7–8% allele frequency and increased to 44–49%, and a presumed de novo mutation in *ampD* increased to 19%, all over 11 days. Conversely, two independent *mexR* mutations conferring levofloxacin resistance went to extinction in <5 days. Altogether, our findings show that low-frequency resistance mutations can expand or contract over large magnitudes within days, suggesting that RETRA-Seq could be utilized during acute infection to accurately survey the in vivo dynamics of resistance mutations.

### Relating dynamics of low-frequency resistance mutations with antibiotic therapy

The expansion and contraction of low-frequency resistance mutations coincided with changes in antibiotic therapy. In several patients, population-wide resistance to β-lactams—cefepime, ceftazidime, piperacillin-tazobactam—changed significantly over time (Fig. 4f, Supplementary Fig. 4; two-sided Mann–Whitney U-test). Furthermore, increased cefepime resistance over time was associated with longer duration of β-lactam therapy, which was calculated as the fraction of days between serial samples that patients were treated with at least one β-lactam (Fig. 4g, Pearson’s *r* = 0.936, *P* = 0.002), that were driven in part by expansions of low-frequency mutations (patients D, F*, G*; Fig. 4b-d). Of note, *oprD* mutants may have emerged after meropenem therapy^10^, which was administered to patient D one day prior to mutant detection (Fig. 1b).

Conversely, changes in therapy also coincided with the contraction of resistance mutations. Patient I* was treated with ceftazidime for several days before day 1 but not during the study period (Supplementary Fig. 1), which coincided with a decrease in cefepime resistance over time (Fig. 4g; Supplementary Fig. 4d, f). In the case of the aforementioned extinction of levofloxacin-resistant *mexR* mutations in patient F* (Fig. 4d), the patient had received ciprofloxacin 6 months earlier but was not treated with fluoroquinolones during the study period. Altogether, our findings show that population resistance can shift within days based on prior and ongoing choice of antibiotic therapy, in part by the expansion or contraction of low-frequency resistance mutations.

## Discussion

Our study shows that the frequencies of resistance mutations change rapidly with antibiotic therapy, highlighting a potential for deep sequencing-guided, short-term cycling of antibiotics within patients as a possible future therapeutic strategy. As resistance mutations can persist in the population for months following treatment^42^, monitoring low-frequency mutations by deep population profiling can inform which antibiotics should be avoided, or conversely, should be actively used in the case of compounds that select against a specific type of resistance^43,44^. While antibiotic cycling has been proposed as a strategy to limit the selective advantage of resistance mutations based on mathematical modeling and experimental evolution studies^45–49^, to date, there are limited data on its clinical efficacy^50^. Our study suggests a possible approach in acute infections, by cycling drugs over days within individual patients over short timescales, which requires further study.

To inform patient-specific antibiotic cycling strategies, molecular diagnostics that deeply and accurately monitor pathogen diversity throughout infection, particularly at the start of infection, are needed. Current culture-based clinical microbiology practice risks missing low-frequency resistant variants^51^. Furthermore, culture-based assays introduce growth bias that differs from the native context of the human lung, where spatial selection is known to occur on pathogens across different niches^8^. Specific alleles encoding resistance could be detected with next-generation molecular assays, e.g., CRISPR-based diagnostics^52^. To monitor known hotspots of mutated genes, we propose resistance-targeted deep amplicon sequencing (RETRA-Seq), using primers that are designed to be suitable across multiple strains, as a highly sensitive method to monitor numerous loci across pathogen genomes.

## Methods

### Patient enrollment

The clinical research conducted in this study complies with all relevant ethical regulations, and the study protocol was approved by the Institutional Review Board of Boston Children’s Hospital. Informed consent was obtained for sample use/collection and medical record review. For paediatric patients, consent was obtained from legal guardians of each patient. Mechanically ventilated patients in the pediatric ICU (via endotracheal tube (ETT) or tracheostomy tube (trach)) were enrolled in the study at the time of suspected infection, defined as when respiratory samples (sputum obtained via endotracheal aspirate or trach aspirate) were ordered by the clinical team for evaluation of suspected infection, with subsequent confirmation of *P. aeruginosa* growth in the clinical microbiology lab. Patients typically experienced fever or hypothermia, increase in ventilator settings or oxygen requirement, and/or increase in quantity and/or change in color or thickness of respiratory secretions (Supplementary Table 1). Patients were classified as having pneumonia if they met these criteria and there was a new and persistent infiltrate on chest radiograph (CXR). Patients were classified as tracheitis if CXR showed no evidence of pneumonia but sputum obtained via ETT aspirate or tracheal aspirate showed few, moderate, or abundant polymorphonuclear leukocytes (PMN) on Gram stain. None of the patients met criteria for a ventilator-associated event (VAE). None of the patients had bacteremia, and all recovered from their infection.

### Sample collection

Sputum and stool samples were processed within 24–48 h of collection from the patient, and solubilized with 10 mM dithiothreitol, frozen in 15% glycerol, and stored at −80 °C until further processing.

### Whole-genome sequencing of *P. aeruginosa* isolates

solates were cultured from sputum and stool samples as previously described^8^. Serial dilutions (10^0^–10^−4^) of each sample in PBS were plated onto cetrimide agar (BD) to identify a dilution plate with growth of 50–300 colonies in total to use for colony picking in order to maximize diversity while minimizing competition between isolates. Colonies (24) were randomly picked by taping a paper pre-marked with 24 random “x” marks to the back of each Petri dish using a clean toothpick, which were placed into 1 mL of LB broth in 96 deep-well plates, then grown overnight at 37 °C with shaking. Half of the saturated cultures were used to make glycerol stocks and the rest were used for DNA extraction (Invitrogen PureLink Pro 96 Genomic DNA Purification Kit). Sequencing libraries of the genomes were prepared as previously described^53^ and sequenced using paired-end 100 bp reads on the Illumina HiSeq 2000 platform, targeting an average sequencing coverage of 40X per isolate.

### Constructing patient-specific reference genomes with long reads

A single colony was isolated from a cetrimide agar plate streaked with each patient’s day 1 sputum sample, grown overnight at 37 °C, and cultured overnight in LB broth with shaking, from which genomes were extracted (Invitrogen PureLink Pro 96 Genomic DNA Purification Kit). Genomes were sequenced on both the PacBio platform (long reads) and on the Illumina HiSeq 2500 platform (short reads) to enable error-correction of assembled contigs. Illumina reads were filtered (min Phred score 15) then trimmed for adapter sequences and assembled de novo using Newbler (v2.7), with minimum contig size 100 bp and minimum coverage at 50×. PacBio reads were assembled de novo using default HGAP 2.0/HGAP 3.0 parameters in the SMRT Analysis Portal (v. 2.3.0). Overlapping contig ends were removed to circularize individual PacBio contigs, and Illumina data was mapped to circularized contigs to detect/correct errors. Comparative genomic analyses were performed using Geneious^54^.

### Constructing a pangenome of coding sequences across reference genomes

A pangenome of all coding sequences found across the patient reference genomes, and two published strains PAO1 and PA14, was constructed with Roary^55^ 3.8.0 (-i 80; minimum percentage identity for blastp). Serotypes were predicted using the web server of PAst^56^.

### Identifying within-patient mutations and short indels

Short reads (Illumina platform) of individual isolate genomes were adapter trimmed (cutadapt v1.8.3), filtered (sickle, quality cutoff 25, length cutoff 50), and aligned to the corresponding patient-specific reference genome (bowtie2 v2.2.4 paired-end, maximum fragment length 2000 bp, no-mixed, dovetail, very-sensitive, n-ceil 0, 0.01). Within-patient single nucleotide polymorphisms (SNPs) were determined by first identifying variant positions of individual isolates with respect to patient-specific references (SAMtools v1.3^57^, FQ ≤ −30), combining the list of variant positions across all isolates of a patient, which were then filtered to high-quality SNP positions. High-quality SNPs were defined as nucleotides at which any two isolates disagreed in the called nucleotide, with both calls meeting a patient-specific FQ threshold that was set based on the distribution of all FQ scores within each patient^2^. Short insertions and deletions (indels) were identified with platypus^58^ (v0.8.1, getVariantsFromBAMs = 1, genSNPs = 0, genIndels = 1, minMapQual = 30), using a QD (ratio of variant quality to read depth) threshold set for each patient based on the distribution of all QD values. All short indels were confirmed by visual inspection of the aligned reads. A genotype matrix (isolates by positions) based on SNPs and indels were constructed for each patient’s pathogen population used for downstream analysis.

### Within-patient phylogenetic trees

A maximum parsimony phylogenetic tree was constructed for each patient, using the genotype matrix of within-patient SNPs and indels, with dnapars v3.696 (PHYLIP package)^59^. Indels were treated as a mutational event, with “I” or “D” designating an insertion or deletion. To root the tree, an “Outgroup” for each patient was created by using the most likely ancestral nucleotide state at each polymorphic locus; this was identified by querying a 101 bp sequence (50 bp upstream and downstream from each mutated locus) against all *Pseudomonas aeruginosa* genomes in the NCBI database with BLASTN. For all polymorphic loci, only one state was found in the database, which was designated as the ancestral state based on its prior observation, while the other state was interpreted as a de novo mutation. All phylogenetic trees were plotted with Toytree v2.0.1^60^.

### Estimating patient colonization time

Bayesian phylogenetic analysis (BEAST 1.10.4^61^) was conducted on the genotype matrix of each patient to estimate the time to the ancestral node in days. Input files were generated with BEAUTi v.10.4, and BEAST 1.10.4 was run under a tree prior of coalescent expansion growth model and otherwise default parameters. Analyses were run using CIPRES^62^.

### Pathway analysis of day 1 mutations

Mutations within day 1 pathogen populations across all patients that were found in annotated coding genes (50 of 81 mutations total) were used to identify associated KEGG pathways on The Pseudomonas Genome Database^63^.

### Twitching motility assay

Assay was conducted as previously reported^64^. Frozen isolates were streaked onto LB-agar plates and grown at 37 °C o/n. Individual colonies were selected with a toothpick and stabbed to the bottom of the twitching assay plate (1% tryptone (Sigma–Aldrich), 0.5% yeast extract (Sigma–Aldrich), 0.5% NaCl (Sigma)); plates were incubated at 37 °C for 20 h. Agar was carefully removed, then plates were stained with 0.1% of Crystal Violet (Sigma) in DI water for 15 min and rinsed with DI water once, then dried. The diameter of the circle was measured in cm.

### Permutation test for shift in < *d*_*MRCA*_ > over time

The distance to the most recent common ancestor (*d*_*MRCA*_), inferred by the maximum parsimony tree of each patient, was calculated for each isolate within a patient population. Mean <*d*_*MRCA*_> of each sputum sample, <*d*_*MRCA*_> ^*t1*^ for day 1 and <*d*_*MRCA*_> ^*t2*^ for follow-up sputum, was calculated within each patient. To test whether the observed difference in means, <*d*_*MRCA*_> ^*t2*^ − <*d*_*MRCA*_> ^*t1*^ was significant, we constructed a null model by permuting the sputum sample assignment across all sputa isolates and recalculating the difference in means across 1000 permutations, from which a one-tailed p-value was calculated.

### Pro-Q gel for lipopolysaccharide

Colonies from an overnight grown Luria Agar plate were resuspended in Luria Broth, normalized to an OD_600_ of 2.0, then pelleted. LPS was prepared as previously documented^65^, and 15 µL of each LPS sample was loaded into each well, then separated by SDS-PAGE in a 10% Mini-PROTEAN TGX gel (Bio-Rad) along with CandyCane glycoprotein ladder (Thermo Fisher). LPS was stained using Pro-Q Emerald 300 LPS Gel Stain (Thermo Fisher) according to the manufacturer’s instructions with slight modifications (the initial fixation step was repeated twice and each washing step was repeated three times).

### O6 serotype Western blot

Colonies from an overnight grown Luria Agar plate were resuspended in Luria Broth, normalized to an OD_600_ of 2.0, then pelleted. LPS was prepared as previously documented^65^, and 15 µL of each LPS sample was loaded into each well, then separated by SDS-PAGE in a 10% Mini-PROTEAN TGX gel (Bio-Rad) along with Precision Plus All Blue Protein ladder (Bio-Rad). The LPS was then transferred to a PVDF membrane and blocked for 1 h, at room temperature, in PBST-5% milk. O6 primary antibody was incubated in a 1:2500 dilution (Group G, Accurate Chemical & Scientific) in PBST-3% BSA overnight at 4 °C. Secondary α-rabbit-HRP IgG (Sigma) was incubated in a 1:10,000 dilution in PBST-3% BSA for 1 h at room temperature. Blot was visualized using Pierce ECL Western Blotting Substrate (Thermo) according to the manufacturer’s instructions.

### Serum killing assay

Isolates were streaked onto TSA plates and incubated at 37 °C o/n, then resuspended in 10 mL PBS + (PBS, 1% proteose peptone, 1 mM CaCl_2_, 1 mM MgCl_2_) to an OD_600_ of 0.25, and diluted 1:23 fold to a final concentration of 5 × 10^5^ CFU/100 µL. Hundred microliters of the diluted culture was mixed with 50% serum (Human Serum, male AB plasma, Sigma–Aldrich H4522; diluted 1:2 with PBS+) in a 96-well round bottom plate in triplicate. Serum assay plates were incubated at 37 °C with shaking at 100 r.p.m. for 1 h, then plated onto TSA, incubated at 37 °C o/n, and quantified for colony forming units (CFU). The PAO1 strain was used as a negative control (not serum sensitive) and PAO1 *galU* mutant^66^ was used as a positive control (serum sensitive).

### Swarming motility assay

Swarming assays were performed as previously reported^67^. Swarming medium contained 0.52% agar with M8 medium supplemented with casamino acids (0.5%), glucose (0.2%) and MgSO_4_ (1 mM). Swarming plates were inoculated with 2.5 µL of an overnight culture grown in LB at 37 °C. Plates were incubated at 37 °C for 16 h. The “Total Swarm Area” is a measure of the number of pixels calculated using ImageJ by first selecting the swarm area, converting images to grayscale (Image → Type → 8-bit), thresholding the image (converting to a black and white image where swarm area is black), and analyzing the particles in the swarm (the number of pixels).

### Biofilm and Psl assay

Biofilm assays were performed as previously described^68^. Overnight cultures (1.5 µL) were inoculated in 100 µL swarming medium and incubated at 37 °C for 24 h. Plates were then stained with 0.1% crystal violet. Absorbance was read at OD_550_. Psl ELISA was conducted following published methods^69^. Briefly, 96-well flat-bottom ELISA plates were coated with bacteria overnight at 4 °C. Diluted anti-Psl monoclonal antibody^69^ (Cam-003; gift from Antonio DiGiandomenico) was added to PBS + 1% BSA (PBS-B)–blocked plates for 1 h, washed with PBS supplemented with 0.1% Tween 20 (PBST), and treated with alkaline phosphatase-conjugated anti–human IgG secondary antibodies (Sigma #A1543) at 1:1000 for 1 h, followed by development with PNP substrate (Sigma).

### AlgD promoter activity assay

Strains carrying the *lacZ* fusion were streaked on PIA or PIA supplemented with 0.1 mM uracil at 37 °C for 24 h. The colonies were then scraped into 4 mL 1× PBS and then diluted to OD_600_ 0.3–0.7. Triplicates of 100 µL of the sample were added to 900 µL of Z-Buffer and 20 µL toluene in a 1.5 mL elution tube. After mixing by inverting 4–5 times tubes were placed with tops open in a shaking incubator at 37 °C for 40 min. After, 200 µL of ortho-Nitrophenyl-β-galactoside (ONPG) (4 mg/mL) (Thermo Scientific, Waltham, MA) was added and the time of color change was recorded the reaction was stopped by adding 500 µL of 1 M Na_2_CO_3_ (Fisher Scientific, Waltham, MA) after 20 min. OD_420_ and OD_550_ were measured using a SpectraMax i3x (Molecular Devices, Downingtown, PA) plate reader. Miller units were calculated using the following formula: *1000 × [OD*_*420*_ *− (1.75* × *OD*_*550*_*)]/[color change time (min.)* × *Sample volume* × *OD*_*600*_*]*. In-frame deletion of *kinB* in strain PA14 was conducted using pEX100T-NotI-Δ*kinB* through a two-step allelic exchange procedure^70^. Single-crossover merodiploid strains were selected based on sensitivity to sucrose (*sacB*) and resistance to carbenicillin. Selected merodiploid strains were then grown in LB broth at 37 °C. Double-crossover strains were selected based on sensitivity to carbenicillin and confirmed through PCR amplification of the flanking region of target gene.

### Antibiotic susceptibility measurements

Minimum inhibitory concentrations (MICs) or zones of inhibition were measured for each isolate in the Infectious Diseases Diagnostic Laboratory at Boston Children’s Hospital, using the Vitek-2 instrument (liquid culture assay) or disk diffusion assay, respectively.

### Preparation of amplicon sequencing library

Total genomic DNA was extracted from each sputum following previously published methods^71^. Briefly, sputum was mixed with 1 mM dithiothreitol (DTT), incubated at 30 °C for 30 min with 0.18 mg/mL lysostaphin and 3.6 mg/mL lysozyme. DNA was purified using the High Pure PCR Template Preparation Kit (Roche) according to the manufacturer’s instructions and eluted in 30 µL of sterile water. A two-step PCR reaction was used to amplify select loci and add adapter sequences as previously documented^72^. *First PCR*. PCR mix was the following: 2 µL DNA template, 10 µL Q5 Hot-Start High-Fidelity 2X Master Mix, 1 µL (NEB #M0494S), 1 µL locus-specific forward primer with UMIs, 1 µL locus-specific reverse primer with UMIs (primers in Supplementary Data 3), 6 µL PCR grade sterile water. Cycling program: hot-start 30 s at 98 °C, 20× cycles of [10 s at 98 °C, 15 s at 67 °C, 15 s at 72 °C], then final extension 2 min at 72 °C. Dilute PCR1 products 1:10 in PCR grade water. *Second PCR*. PCR mix was the following: 2 µL 1:10 diluted PCR1 product, 10 µL Q5 Hot-Start High-Fidelity 2X Master Mix, 1 µL universal forward primer, 1 µL sample-specific barcoded reverse primer, 6 µL PCR grade sterile water. Cycling program: hot-start 30 s at 98 °C, 20× cycles of [10 s at 98 °C, 30 s at 72 °C], then final extension 2 min at 72 °C. Pool and clean up PCR reaction using a column (Zymo Research #D4013). Amplicon libraries were assessed for correct fragment sizes (350–400 bp) on a 2% agarose gel and quantified using Qubit. Libraries were sequenced on a MiSeq v2 300 cycle kit (Illumina #MS-102-2002) with Read 1: 150 cycles, Index 1: 8 cycles, Read 2: 150 cycles, sequenced at a minimum saturating depth defined as 1/Illumina sequencing error rate, estimated as 0.5%^73^.

### Analysis of amplicon sequencing data

Paired-end reads were trimmed for adapter sequences and filtered with cutadapt (pair-filter q30), then merged across overlapping regions of Read 1 and Read 2 with vsearch v2.15.2, and aligned to the coding sequence of mutated genes (bowtie2–local). From each merged and aligned read, we extracted both the sequence at the profiled locus (wild-type vs. mutant) and the unique UMI sequence (from both forward and reverse), which were used to count the number of unique UMI corresponding to each allele type. Uncertainty of each allele frequency was calculated using the Wilson Score interval based on UMI counts using the statsmodels package (proportion_confint).

### Mapping mutations onto protein structure

Protein sequences of mutated genes were queried in the Protein Data Bank (PDB) to find the closes homolog structures: NalD (PDB ID: 5DAJ, 94% identity), AnmK (3QBW, 99% identity), MexR (1LNW, 99% identity), AmpR (5MMH, 100% identity), PA0810 (3UMC, 93% identity).

### Statistical analyses

Statistical analyses using Mann–Whitney U-test (ranksum) and Kolmogorov–Smirov test (kstest2) were conducted using built-in packages in MATLAB (R2017b). ANOVA tests for phenotype assays were conducted in Prism (GraphPad). Permutation test for <*d*_*MRCA*_>  were conducted in python, with code available at GitHub https://github.com/hattiechung/Paeruginosa_acute_infection.

### Reporting summary

Further information on research design is available in the Nature Research Reporting Summary linked to this article.

## Supplementary information

Supplementary Information

Description of Additional Supplementary Files

Supplementary Data 1

Supplementary Data 2

Supplementary Data 3

Reporting Summary

## Data Availability

The patient-specific reference genomes constructed from PacBio sequencing in this study have been deposited to Sequence Read Archive (SRA) under accession code PRJNA638217. The raw FASTQ files of Illumina sequencing of the 420 isolates generated in this study have been deposited to SRA under accession code PRJNA622605. The list of all within-patient pathogen variants is available in Supplementary Data 1. The processed data of genomic variants used to construct phylogenetic trees and the data on antibiotic resistance susceptibility profiles of all 420 isolates are available on GitHub [https://github.com/hattiechung/Paeruginosa_acute_infection]. Protein structure data are available at the Protein Data Bank under the following IDs: 5DAJ, 3QBW, 1LNW, 5MMH, 3UMC. Source data are provided with this paper.

## Source data

Source Data

## Materials availability

All 420 clinical isolates of *P. aeruginosa* used for whole-genome sequencing on the Illumina platform and for antibiotic susceptibility measurements are available upon request. Source data are provided with this paper.

## Code availability

Code used for analyses are available on GitHub [https://github.com/hattiechung/Paeruginosa_acute_infection].
