# Supplementary material for: Rapid expansion and extinction of antibiotic resistance mutations during treatment of acute bacterial respiratory infections
Source: Nat Commun. 2022 Mar 9;13:1231. doi: 10.1038/s41467-022-28188-w (PMC8907320; doi:10.1038/s41467-022-28188-w)
Supplement: Supplementary file 1 — Supplementary Information [file 41467_2022_28188_MOESM1_ESM.pdf]

Rapid expansion and extinction of antibiotic resistance mutations during treatment of acute bacterial respiratory infections

Supplementary Information

- Supplementary Table 1
- Supplementary Figures 1-5

Supplementary Table 1. Clinical attributes of patient cohort.

Patient	Age (approx., years)*	Chronic conditions	*P. aeruginosa* history	Time from 1st *P. aeruginosa* infection to Day 1 sample	Time from most recent *P. aeruginosa* infection to Day 1 sample	Type of artificial airway at Day 1 sample	Duration of any artificial airway before Day 1 sample	Duration of tracheostomy before Day 1 sample	*P. aeruginosa* load on Day 1 sample[1]	PMN on Day 1 sample[2]	Infection type	Days of ventilation after Day 1[3]
A	2-5	Lysosomal storage disease, cardiomyopathy	New	N/A	N/A	Tracheostomy	6 months	6 months	Few	Abundant	Pneumonia and RSV	50
B	0.5-2	Epilepsy, brain malformation (Dandy Walker)	New	N/A	N/A	ETT	1 day	N/A	Moderate	Moderate	Pneumonia	>300
C	0.5-2	Trisomy 21, congenital heart disease, tracheomalacia	New	N/A	N/A	Tracheostomy	6 weeks	3 weeks	Few	Few/ Moderate[5]	Tracheitis	>265
D	2-5	Congenital heart disease	New	N/A	N/A	ETT	3 days	N/A	Abundant	Moderate	Tracheitis	11
E	0.08-0.5	Repaired esophageal atresia and tracheoesophageal fistula	Prior[4]	<2 months	11 days	ETT	3 weeks	N/A	Moderate	Moderate	Tracheitis	14
F	>21	Congenital heart disease	Prior	Unknown	19 days	Tracheostomy	5 weeks	2 weeks	Moderate	Few	Tracheitis	17
G	2-5	Pulmonary hypertension, immunodeficiency	Prior	2 years	5 weeks	Tracheostomy	10 weeks	7 weeks	Abundant	Abundant	Tracheitis	>7
H	2-5	Multiple congenital anomalies, congenital heart disease, hypoxic ischemic encephalopathy	Prior	3 months (estimated) up to 4 years	Unknown	Tracheostomy	12 weeks	9 weeks	Abundant	Moderate	Tracheitis (2nd sample)	16
I	6-12	Epilepsy, brain malformation (lissencephaly)	Prior	6 years	7 months	ETT	1 day	N/A	Moderate	Moderate	Tracheitis and RSV	13

Abbreviations: ETT, endotracheal tube; PMN, polymorphonuclear leukocyte. *55% female. [1]For semiquantitative culture results, abundant is approximately ≥10^6 CFU/mL; moderate is ≥10^5 CFU/mL, few is ≥10^4 CFU/mL, and rare is ≥10^3 CFU/mL. [2]For semiquantitative Gram stain results, abundant = >25, moderate =10-25, few = 1-9, and rare = <1 PMN per low power field. [3]Days of ventilation were not available for some patients transferred to other hospitals (listed as >x days). [4]Presumed (culture at outside hospital 11 days prior to 1st sample grew mixed bacteria, not speciated). [5]Moderate PMN on sample sent 4 days later.

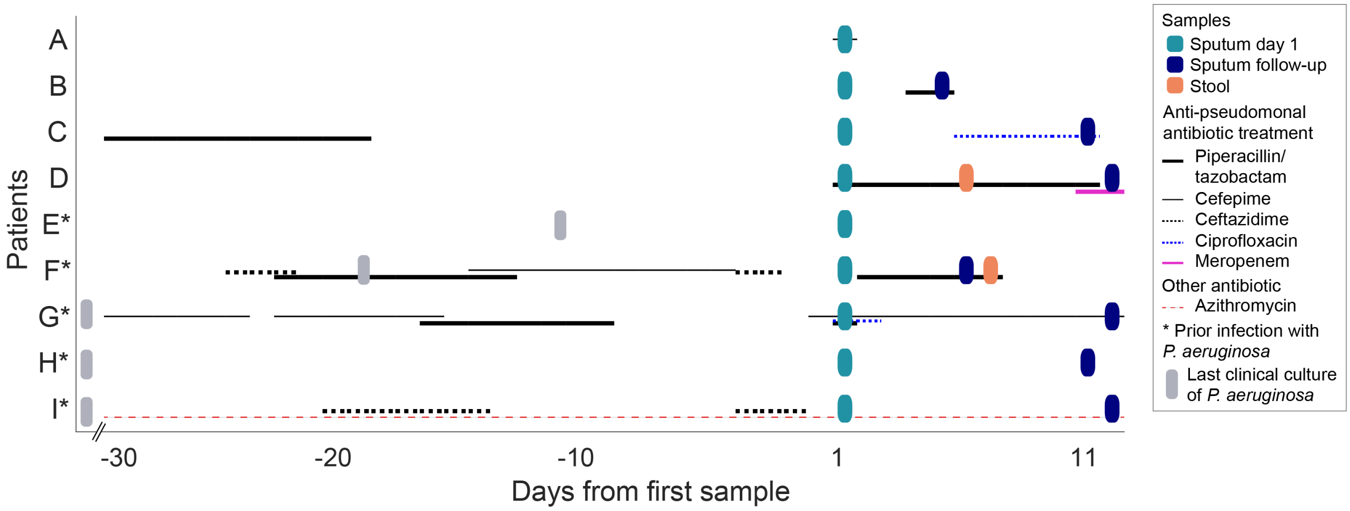

Supplementary Figure 1. Extended antibiotic treatment history of patients. Patient samples collected over time (x axis) from the onset of symptoms (day 1), as in **Fig. 1b**, showing sputum (day 1 in teal, follow-up in navy blue) and stool (tan). Asterisk (*) denotes patients with documented history of *P. aeruginosa* infection. Horizontal lines indicate days of treatment with anti-pseudomonal and other antibiotics shown from 30 days before the first sample within each patient. In patients with prior *P. aeruginosa* infection, the most recent documented clinical culture of *P. aeruginosa* is shown by a gray box; documentation more than 30 days before day 1 are shown to the left of the breakpoint (hatched black tracks, x axis). Antibiotics: Piperacillin/tazobactam (weighted black), cefepime (thin black), ceftazidime (dotted black), ciprofloxacin (dotted blue), meropenem (weighted pink), azithromycin (dashed red).

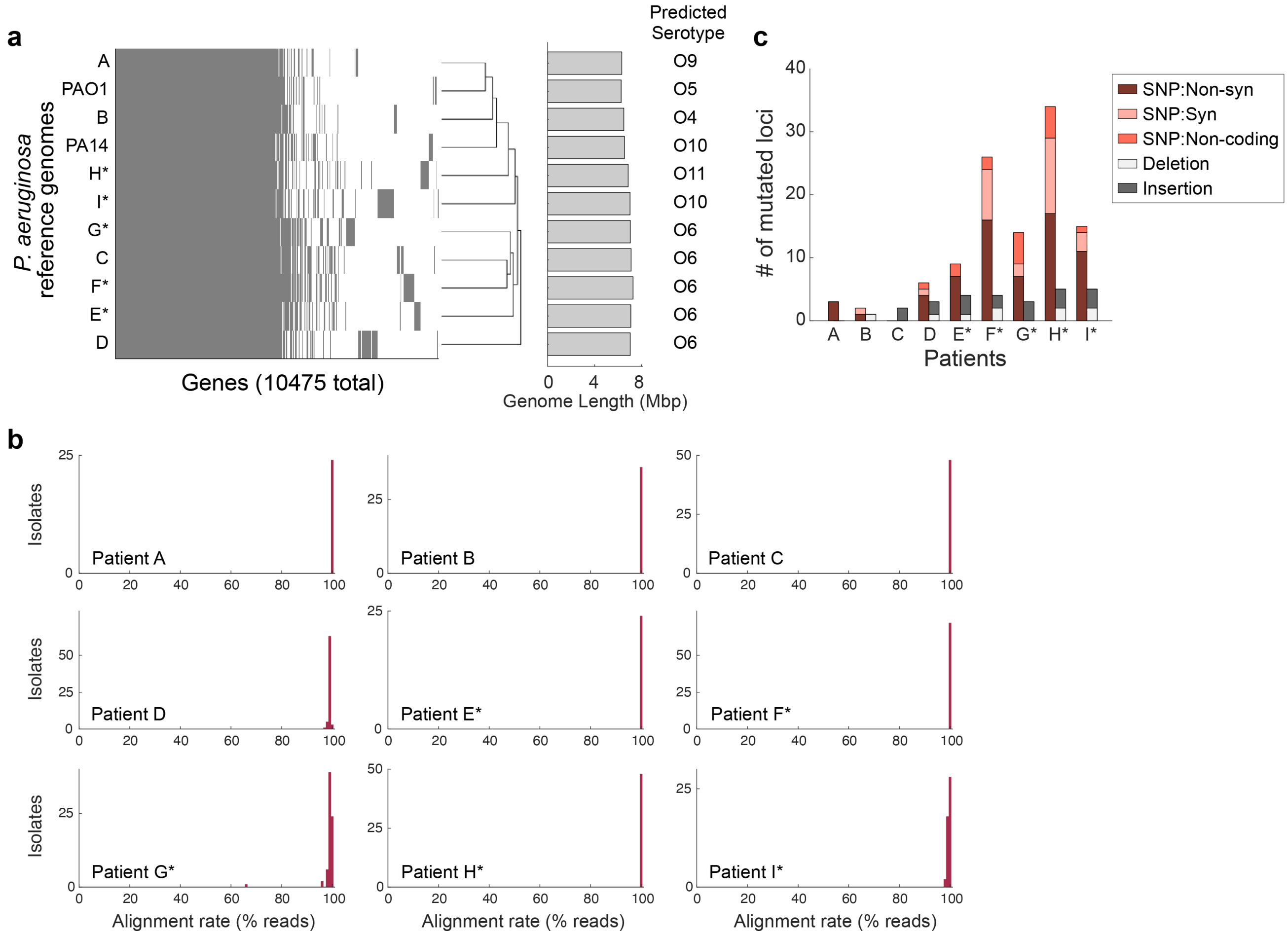

Supplementary Figure 2. Maximizing the identification of within-patient polymorphisms by constructing patient-specific reference genomes. a. Clustermap of all genes of the pangenome constructed across the patient strains and two reference strains, PAO1 and PA14, showing the presence (gray) or absence (white) of coding genes (x axis; 10,475 genes total) in each reference genome (y axis). *Middle*: Genome length of constructed reference genome in units of mega-base pairs (Mbp). *Right*: Serotypes of each strain predicted *in silico*. **b.** Distribution of alignment rates across isolates, calculated as the percentage of short-reads from whole-genome sequencing of individual isolates aligned to patient-specific reference genomes. **c.** Distribution of the number of polymorphic mutation types (y axis) within each patient's population (x axis), shown by subtypes of single nucleotide polymorphisms (left bar: non-synonymous in maroon, synonymous in rose, non-coding in salmon) and subtypes of short indels (right bar: deletions in light grey, insertions in dark grey).

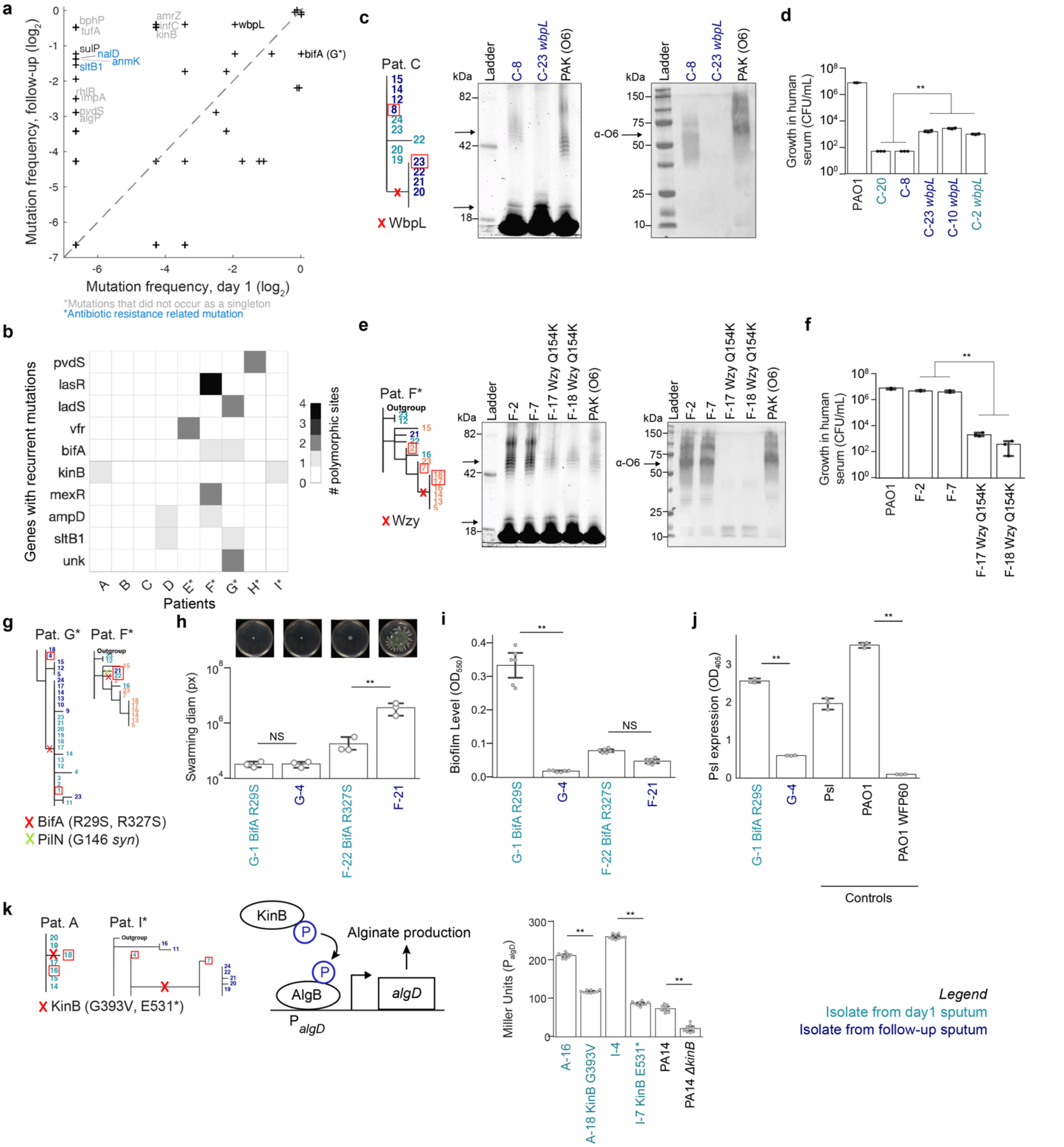

a
Mutation frequency, follow-up (log2)
Mutation frequency, day 1 (log2)
bphP
tufA
amrZ
infC
kinB
wbpL
sulP
nalD
anmK
sltB1
bifA (G*)
rhlR
impA
pvdS
algP
*Mutations that did not occur as a singleton
*Antibiotic resistance related mutation
b
Genes with recurrent mutations
pvdS
lasR
ladS
vfr
bifA
kinB
mexR
ampD
sltB1
unk
Patients
polymorphic sites
c
Pat. C
X WbpL
Ladder
C-8
C-23 wbpL
PAK (O6)
kDa
α-O6
d
Growth in human serum (CFU/mL)
PAO1
C-20
C-8
C-23 wbpL
C-10 wbpL
C-2 wbpL
e
Pat. F*
Outgroup
X Wzy
F-2
F-7
F-17 Wzy Q154K
F-18 Wzy Q154K
f
g
Pat. G*
Pat. F*
X BifA (R29S, R327S)
X PilN (G146 syn)
h
Swarming diam (px)
NS
G-1 BifA R29S
G-4
F-22 BifA R327S
F-21
i
Biofilm Level (OD550)
j
Psl expression (OD405)
Psl
PAO1
PAO1 WFP60
Controls
k
Pat. A
Pat. I*
X KinB (G393V, E531*)
KinB
P
Alginate production
AlgB
algD
PalgD
Miller Units (PalgD)
A-16
A-18 KinB G393V
I-4
I-7 KinB E531*
PA14
PA14 ΔkinB
Legend
Isolate from day1 sputum
Isolate from follow-up sputum

Supplementary Figure 3. Clinically relevant phenotypes of mutant isolates compared near-isogenic controls. a. Comparing the frequency of each mutation (points) observed in the pathogen population at day 1 (x axis) vs. in follow-up (y axis) sputum, based on the fraction of cultured isolates. Dotted gray line, *y=x*. Annotations indicate mutations that occurred in coding genes at >5% frequency in at least one time point (blue: antibiotic resistance associated mutations as in **Fig. 3**; gray: mutations that did not occur as a singleton). **b.** Genes with recurrent mutations (rows), defined as those with two mutated polymorphic positions or more (color, grayscale), within or across patients (columns). **c-f.** Mutations disrupting lipopolysaccharide (LPS) and O antigen presentation (**c,e**) lead to altered sensitivity to human serum (**d,f**). **c,e.** *Left*: Inset of phylogenies (as in **Fig. 3**) showing mutant and isogenic control isolates (red box) used for phenotyping, which are separated by the singleton mutation marked on the branch (red x). Characterizing mutants of WbpL (single nucleotide frameshift deletion in the O antigen glycosyltransferase) in Patient C (**c**) or Wzy (non-synonymous substitution in a homolog of the O-polysaccharide polymerase) in Patient F* (**e**). **c-f.** Isolates: controls (C-8 in **c**; F-2 and F-7 in **e**), mutants (C-23 in **c**; F-17, F-18 in **e**), and *PAK* reference strain (serotype O6). Ladder indicates size (kDa). *Middle:* LPS gel stain image (Pro-Q Emerald 300) showing truncated LPS banding patterns (rows) in mutant isolates compared to controls (columns). Top and bottom arrows indicate larger and truncated LPS banding patterns, respectively. *Right*: Western blot detection of O antigen with anti-O6 antibody, showing intact recognition in controls (arrow) but absence in mutants. **d,f.** Altered sensitivity to human serum in mutants with disrupted O-antigen. Isolates (x axis) assayed for growth in human serum (CFU/mL, y axis), 3 technical replicates (dots); representative of 3 biologically independent replicates. Bars show median; error bars, standard error. Adjusted *P* values, Tukey's multiple comparisons test between C-20 or C-8 *vs* C-23 or C-10 or C-2 ($P<0.001$); F-2 or F-7 *vs* F-17 or F-18 ($P<0.001$). **g-j.** Phenotypic impact of BifA mutations. **g.** Inset of phylogenies (as in **Fig. 3**, left: patient G*, right: patient F*) showing mutant and control isolates (red box) separated by KinB mutations labeled on the branch (red x; R29S singleton in Patient G*, R327S in patient F*). Isolate used as control in Patient F* harbored an additional synonymous

mutation (G146) in the gene PilN. **h-j.** Control (G-4, F-21) and mutant (G-1 R29S mutant, F-22 R327S mutant) isolates (x axis) were phenotyped for swarming (**h**, diameter in corresponding images, pixels, y axis), biofilm production (**i**, OD_{550}, y axis), and Psl expression measured by ELISA (**j**, OD_{405}, y axis), each across 3, 6, or 3 technical replicates, respectively. Bars show median, error bars show standard error. Adjusted *P* values, Tukey's multiple comparisons test, from left to right (**h-j**): **P*=0.0045 (**h**), ***P*<0.001 (**i**), ***P*<0.001 (**j**). NS – not significant. **k.** Phenotypic impact of KinB mutations. Inset of phylogenies (as in **Fig. 3**, left: patient A, right: patient I*) showing mutant and control isolates, red box (controls: A-16, I-4; mutants: A-18 G393V mutant, I-7 E531* mutant). KinB phosphorylates AlgB, which regulates *algD* and subsequent alginate production. Bar graph: isolates (x axis) have altered *algD* promoter activity (Miller units of β-gal expression, y axis); bars show median, error bars show standard error, for 8 technical replicates. ***P*<0.001, adjusted *P* value, Tukey's multiple comparisons test.

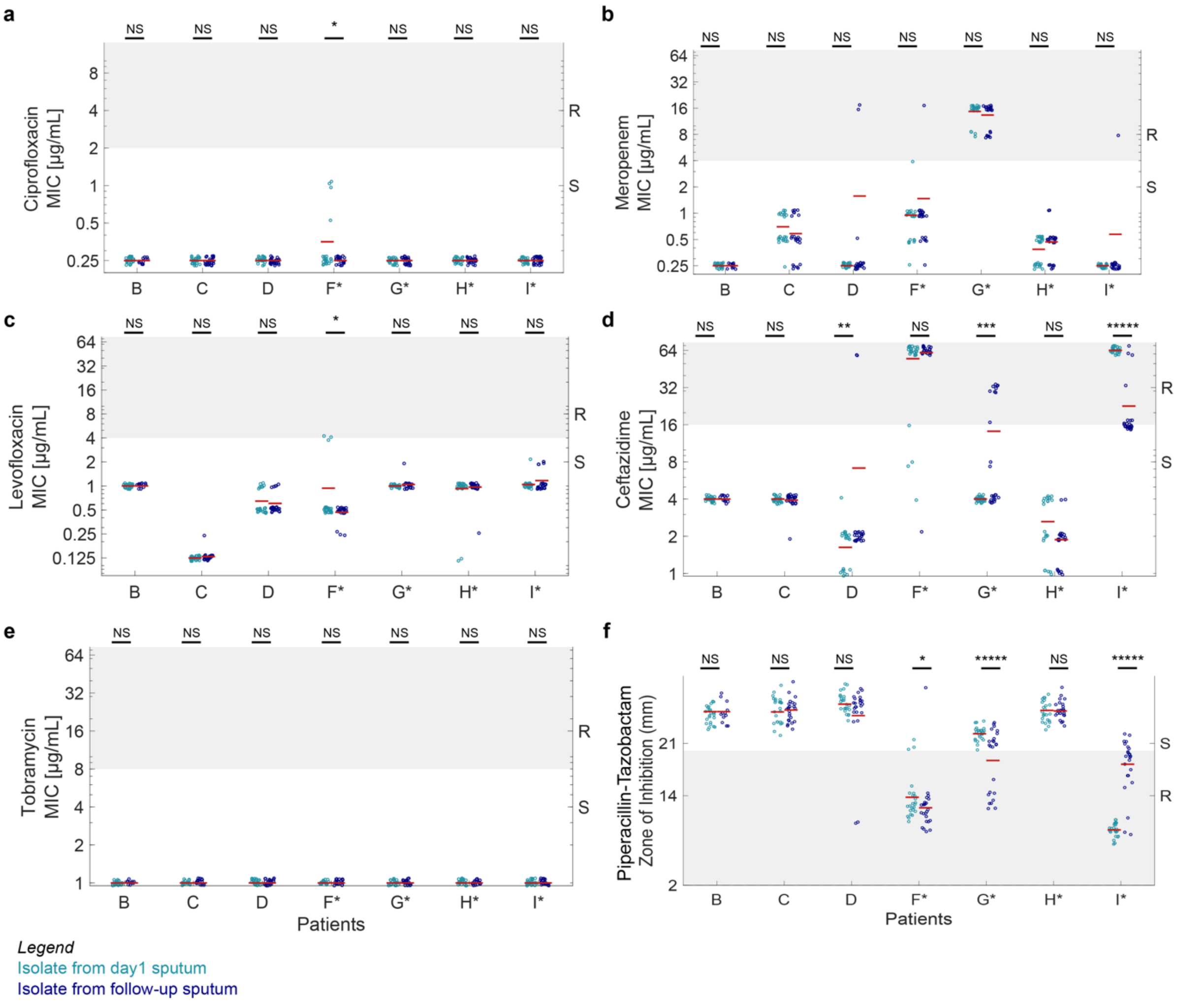

Supplementary Figure 4. Changes in antibiotic susceptibility of sputum isolates across sputa samples. Antibiotic susceptibility of individual isolates (dots, n=24 biologically independent isolates per sputum sample) determined by the minimum inhibitory concentration in liquid cultures (MIC, µg/mL y axis, **a-e**) or by the zone of inhibition via disk diffusion assay (mm, y axis, **f**). Isolates (dots) are shown for day 1 (teal) and follow-up (dark blue) sputum samples. Antibiotic susceptibility regimes indicated on the right and by background color, according to breakpoints defined by the Clinical Laboratory Standards Institute (CLSI), with resistant (R) or intermediate susceptibility in gray and sensitive (S) in white. Significance in difference of means (horizontal red line) across sputum samples within each patient (two-sided Mann-Whitney test), from left to right: *P=0.041 (**a**); *P=0.016 (**c**); **P=0.002, ***P=0.0002, *****$P<10^{-5}$ (**d**); *P=0.018, *****$P=9*10^{-6}$, *****$P<10^{-5}$ (**f**). NS – not significant.

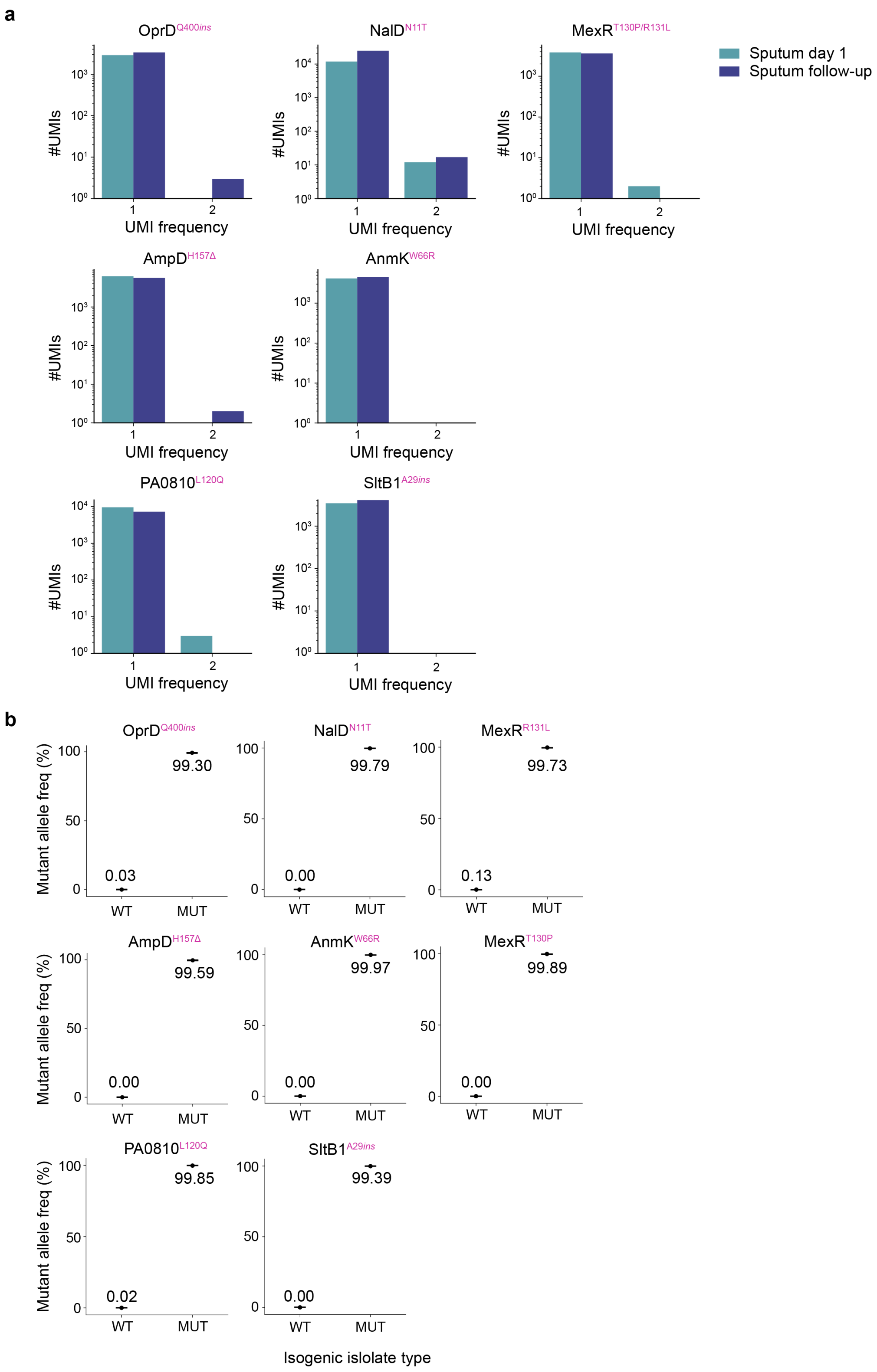
a
OprD Q400ins
NalD N11T
MexR T130P/R131L
Sputum day 1
Sputum follow-up
#UMIs
UMI frequency
AmpD H157Δ
AnmK W66R
PA0810 L120Q
SltB1 A29ins
b
OprD Q400ins
99.30
0.03
NalD N11T
99.79
0.00
MexR R131L
99.73
0.13
AmpD H157Δ
99.59
0.00
AnmK W66R
99.97
0.00
MexR T130P
99.89
0.00
PA0810 L120Q
99.85
0.02
SltB1 A29ins
99.39
0.00
Mutant allele freq (%)
WT
MUT
Isogenic islolate type

Supplementary Figure 5. Detecting unique bacterial genomes corresponding to each allele with RETRA-Seq. a. To account for amplification bias, primers barcoded with unique molecular identifiers (UMIs) were used to amplify total DNA extracted from sputum. Number of distinct UMIs (y axis) found in each amplicon library (individual plots, title) grouped by the frequency of observed for each UMI (x axis) in raw sequencing data of each sputum sample (bar color; teal, day 1 sputum and navy blue, follow-up sputum). **b.** Mutant allele frequencies (y axis; exact frequencies labeled on plot) measured by deep amplicon sequencing in isogenic controls (x axis), left: wild-type (WT) colony, right: mutant colony, plots arranged as in **Fig. 4b-d**. Error bars: Wilson Score interval, calculated over n=10^2-10^4 unique genomes observed per mutant or WT, examined in 1 amplicon sequencing experiment.
