# Supplementary material for: Rapid expansion and extinction of antibiotic resistance mutations during treatment of acute bacterial respiratory infections
Source: Nat Commun. 2022 Mar 9;13:1231. doi: 10.1038/s41467-022-28188-w (PMC8907320; doi:10.1038/s41467-022-28188-w)
Supplement: Supplementary file 2 — Description of Additional Supplementary Files [file 41467_2022_28188_MOESM2_ESM.pdf]

Description of Additional Supplementary Files

File Name: Supplementary Data 1

Description: List of all genomic variants identified within each patient's pathogen population.

File Name: Supplementary Data 2

Description: List of all genomic variants identified with each patient's pathogen population at only Day 1.

File Name: Supplementary Data 3

Description: Primers used for resistance-targeted amplicon sequencing (RETRA-seq).
