# Supplementary material for: Rapid expansion and extinction of antibiotic resistance mutations during treatment of acute bacterial respiratory infections
Source: Nat Commun. 2022 Mar 9;13:1231. doi: 10.1038/s41467-022-28188-w (PMC8907320; doi:10.1038/s41467-022-28188-w)
Supplement: Supplementary file 6 — Reporting Summary [file 41467_2022_28188_MOESM6_ESM.pdf]

nature research

Corresponding author(s): Gregory P. Priebe, Roy Kishony

Last updated by author(s): Jan 3, 2022

Reporting Summary

Nature Research wishes to improve the reproducibility of the work that we publish. This form provides structure for consistency and transparency in reporting. For further information on Nature Research policies, see our Editorial Policies and the Editorial Policy Checklist.

Statistics

For all statistical analyses, confirm that the following items are present in the figure legend, table legend, main text, or Methods section.

n/a	Confirmed	
☐	☒	The exact sample size (*n*) for each experimental group/condition, given as a discrete number and unit of measurement
☐	☒	A statement on whether measurements were taken from distinct samples or whether the same sample was measured repeatedly
☐	☒	The statistical test(s) used AND whether they are one- or two-sided<br>*Only common tests should be described solely by name; describe more complex techniques in the Methods section.*
☒	☐	A description of all covariates tested
☐	☒	A description of any assumptions or corrections, such as tests of normality and adjustment for multiple comparisons
☐	☒	A full description of the statistical parameters including central tendency (e.g. means) or other basic estimates (e.g. regression coefficient) AND variation (e.g. standard deviation) or associated estimates of uncertainty (e.g. confidence intervals)
☐	☒	For null hypothesis testing, the test statistic (e.g. *F*, *t*, *r*) with confidence intervals, effect sizes, degrees of freedom and *P* value noted<br>*Give P values as exact values whenever suitable.*
☒	☐	For Bayesian analysis, information on the choice of priors and Markov chain Monte Carlo settings
☒	☐	For hierarchical and complex designs, identification of the appropriate level for tests and full reporting of outcomes
☐	☒	Estimates of effect sizes (e.g. Cohen's *d*, Pearson's *r*), indicating how they were calculated

Our web collection on statistics for biologists contains articles on many of the points above.

Software and code

Policy information about availability of computer code

Data collection	Illumina HiSeq 2500 software (HCS version 2) was used for DNA sequencing.
Data analysis	Patient-specific reference genomes were assembled de novo from a combination of PacBio reads and Illumina reads using default HGAP 2.0/ HGAP 3.0 parameters in the SMRT Analysis Portal (v. 2.3.0), Newbler 2.7, and Geneious.Pangenome of coding genes was constructed with Roary 3.8.0. Raw FASTQ files of Illumina reads were preprocessed with cutadapt 1.8.3 and sickle 1.3.3, aligned to reference genomes using bowtie2 v2.2.4, and analyzed for SNPs and indels with samtools 1.3 and platypus 0.8.1. Phylogenetic trees were constructed with dnapars v3.696 (PHYLIP package) and plotted with toytree v2.0.1. MATLAB and python code used for further analysis are available on GitHub (https:// github.com/hattiechung/Paeruginosa_acute_infection).

For manuscripts utilizing custom algorithms or software that are central to the research but not yet described in published literature, software must be made available to editors and reviewers. We strongly encourage code deposition in a community repository (e.g. GitHub). See the Nature Research guidelines for submitting code & software for further information.

Data

Policy information about availability of data

All manuscripts must include a data availability statement. This statement should provide the following information, where applicable:

- Accession codes, unique identifiers, or web links for publicly available datasets
- A list of figures that have associated raw data
- A description of any restrictions on data availability

The patient-specific reference genomes constructed from PacBio sequencing in this study have been deposited to Sequence Read Archive (SRA) under accession code PRJNA638217 [https://www.ncbi.nlm.nih.gov/bioproject/PRJNA638217]. The raw FASTQ files of Illumina sequencing of the 420 isolates generated in this study have been deposited to SRA under accession code PRJNA622605 [https://www.ncbi.nlm.nih.gov/bioproject/PRJNA622605]. The list of all within-patient pathogen

variants is available in Supplementary Data 1. The processed data of genomic variants used to construct phylogenetic trees and the data on antibiotic resistance susceptibility profiles of all 420 isolates are available on GitHub [https://github.com/hattiechung/Paeruginosa_acute_infection]. Protein structure data are available at the Protein Data Bank under the following IDs: 5DAJ [https://www.rcsb.org/structure/5DAJ], 3QBW [https://www.rcsb.org/structure/3QBW], 1LNW [https://www.rcsb.org/structure/1LNW], 5MMH [https://www.rcsb.org/structure/5MMH], 3UMC [https://www.rcsb.org/structure/3UMC].

Field-specific reporting

Please select the one below that is the best fit for your research. If you are not sure, read the appropriate sections before making your selection.

☒ Life sciences ☐ Behavioural & social sciences ☐ Ecological, evolutionary & environmental sciences

For a reference copy of the document with all sections, see nature.com/documents/nr-reporting-summary-flat.pdf

Life sciences study design

All studies must disclose on these points even when the disclosure is negative.

Sample size	Patient sample sizes were not calculated a priori. We estimated that 7 serially sampled patients were adequate for this study, as we conducted a deep population survey within each patient.<br><br>We calculated that 24 colonies per sample were adequate with the goal of detecting mutations at >4% frequency.
Data exclusions	There were no data exclusions.
Replication	For characterizing clinically relevant phenotypes of mutants, biological replicates of isolates determined to have the same genotype were used wherever possible.<br><br>All in vitro experiments were performed 2-3 times, and all findings were replicated successfully.
Randomization	We conducted a random sampling of Pseudomonas aeruginosa colonies from each cultured plate of sputum or stool. To ensure random selection of colonies, we marked a piece of paper cut to the size of the Petri dish with 24 "x" marks. We taped this paper to the back of each cetrimide dish and picked the colony closest to each "x" mark.<br><br>As this was an observational study, random allocation of patients is not applicable to this study.<br><br>For characterizing clinically relevant phenotypes of mutants, we allocated isolates as control vs. experimental based on their genotype.
Blinding	Investigators performing laboratory assays were blinded to the patient identification and patient characteristics.

Reporting for specific materials, systems and methods

We require information from authors about some types of materials, experimental systems and methods used in many studies. Here, indicate whether each material, system or method listed is relevant to your study. If you are not sure if a list item applies to your research, read the appropriate section before selecting a response.

Materials & experimental systems

n/a	Involved in the study	
☐	☒	Antibodies
☒	☐	Eukaryotic cell lines
☒	☐	Palaeontology and archaeology
☒	☐	Animals and other organisms
☐	☒	Human research participants
☒	☐	Clinical data
☒	☐	Dual use research of concern

Methods

n/a	Involved in the study	
☒	☐	ChIP-seq
☒	☐	Flow cytometry
☒	☐	MRI-based neuroimaging

Antibodies

Antibodies used	The anti-Psl monoclonal antibody (Cam-003) was a gift from Antonio DiGiandomenico of AstraZeneca (formerly MedImmune, now at AstraZeneca). The anti-O6 antibody is Group G, Accurate Chemical & Scientific (Denka Seiken Co. Ltd. #213648). The anti-human IgG alkaline phosphatase-conjugated secondary is from Sigma (#A1543).
Validation	The anti-Psl antibody was validated in ref. 69. The anti-O6 antibody was validated in this paper on the Pseudomonas aeruginosa PAK strain, which has known O6 serotype (Fig 3d).

Human research participants

Policy information about studies involving human research participants

Population characteristics	Supplementary Table 1 describes the patient characteristics.
Recruitment	Patients who were undergoing mechanical ventilation in the pediatric ICU via endotracheal tube (ETT) or tracheostomy tube (trach) were enrolled in the study when respiratory samples (TT aspirate or trach aspirate) were growing P. aeruginosa in the clinical microbiology lab had been ordered by the clinical team for evaluation of suspected infection, which typically included fever (or hypothermia), increase in ventilator settings or oxygen requirement, and/or increase in quantity and/or change in color or thickness of respiratory secretions.<br><br>We note that our recruitment and selection criteria of patients for this study would be biased against patients with mild infections whose symptoms resolved too quickly for us to sample serially.
Ethics oversight	The study was approved by the Boston Children's Hospital IRB (protocol #P00005656), and informed consent was obtained for sample use/collection and medical record review.

Note that full information on the approval of the study protocol must also be provided in the manuscript.
